# 115 Examination of Voice Changes up to Five Years After Burn Injury

**DOI:** 10.1093/jbcr/irae036.114

**Published:** 2024-04-17

**Authors:** Kaitlyn Chacon, Edward Santos, Kara McMullen, Lauren Shepler, Audra Clark, Chiaka O Akarichi, Haig A Yenikomshian, Caitlin M Orton, Carla Tierney-Hendricks, Colleen M Ryan, Jeffrey C Schneider

**Affiliations:** Spaulding Rehabilitation Hospital, Charlestown, MA; University of Washington, Seattle, WA; Spaulding Rehabilitation Hospital, Harvard Medical School, Boston, MA; University of Texas Southwestern, Dallas, TX; University of Texas Southwestern/Parkland Hospital, Dallas, Texas; University of Southern California, Los Angeles, CA; UW Medicine Regional Burn Center, Harborview Medical Center, Seattle, WA; Spaulding Rehabilitation Hospital, Boston, Massachusetts; Massachusetts General Hospital/Shriners Children's, Boston, MA; Spaulding Rehabilitation Hospital, Charlestown, MA; University of Washington, Seattle, WA; Spaulding Rehabilitation Hospital, Harvard Medical School, Boston, MA; University of Texas Southwestern, Dallas, TX; University of Texas Southwestern/Parkland Hospital, Dallas, Texas; University of Southern California, Los Angeles, CA; UW Medicine Regional Burn Center, Harborview Medical Center, Seattle, WA; Spaulding Rehabilitation Hospital, Boston, Massachusetts; Massachusetts General Hospital/Shriners Children's, Boston, MA; Spaulding Rehabilitation Hospital, Charlestown, MA; University of Washington, Seattle, WA; Spaulding Rehabilitation Hospital, Harvard Medical School, Boston, MA; University of Texas Southwestern, Dallas, TX; University of Texas Southwestern/Parkland Hospital, Dallas, Texas; University of Southern California, Los Angeles, CA; UW Medicine Regional Burn Center, Harborview Medical Center, Seattle, WA; Spaulding Rehabilitation Hospital, Boston, Massachusetts; Massachusetts General Hospital/Shriners Children's, Boston, MA; Spaulding Rehabilitation Hospital, Charlestown, MA; University of Washington, Seattle, WA; Spaulding Rehabilitation Hospital, Harvard Medical School, Boston, MA; University of Texas Southwestern, Dallas, TX; University of Texas Southwestern/Parkland Hospital, Dallas, Texas; University of Southern California, Los Angeles, CA; UW Medicine Regional Burn Center, Harborview Medical Center, Seattle, WA; Spaulding Rehabilitation Hospital, Boston, Massachusetts; Massachusetts General Hospital/Shriners Children's, Boston, MA; Spaulding Rehabilitation Hospital, Charlestown, MA; University of Washington, Seattle, WA; Spaulding Rehabilitation Hospital, Harvard Medical School, Boston, MA; University of Texas Southwestern, Dallas, TX; University of Texas Southwestern/Parkland Hospital, Dallas, Texas; University of Southern California, Los Angeles, CA; UW Medicine Regional Burn Center, Harborview Medical Center, Seattle, WA; Spaulding Rehabilitation Hospital, Boston, Massachusetts; Massachusetts General Hospital/Shriners Children's, Boston, MA; Spaulding Rehabilitation Hospital, Charlestown, MA; University of Washington, Seattle, WA; Spaulding Rehabilitation Hospital, Harvard Medical School, Boston, MA; University of Texas Southwestern, Dallas, TX; University of Texas Southwestern/Parkland Hospital, Dallas, Texas; University of Southern California, Los Angeles, CA; UW Medicine Regional Burn Center, Harborview Medical Center, Seattle, WA; Spaulding Rehabilitation Hospital, Boston, Massachusetts; Massachusetts General Hospital/Shriners Children's, Boston, MA; Spaulding Rehabilitation Hospital, Charlestown, MA; University of Washington, Seattle, WA; Spaulding Rehabilitation Hospital, Harvard Medical School, Boston, MA; University of Texas Southwestern, Dallas, TX; University of Texas Southwestern/Parkland Hospital, Dallas, Texas; University of Southern California, Los Angeles, CA; UW Medicine Regional Burn Center, Harborview Medical Center, Seattle, WA; Spaulding Rehabilitation Hospital, Boston, Massachusetts; Massachusetts General Hospital/Shriners Children's, Boston, MA; Spaulding Rehabilitation Hospital, Charlestown, MA; University of Washington, Seattle, WA; Spaulding Rehabilitation Hospital, Harvard Medical School, Boston, MA; University of Texas Southwestern, Dallas, TX; University of Texas Southwestern/Parkland Hospital, Dallas, Texas; University of Southern California, Los Angeles, CA; UW Medicine Regional Burn Center, Harborview Medical Center, Seattle, WA; Spaulding Rehabilitation Hospital, Boston, Massachusetts; Massachusetts General Hospital/Shriners Children's, Boston, MA; Spaulding Rehabilitation Hospital, Charlestown, MA; University of Washington, Seattle, WA; Spaulding Rehabilitation Hospital, Harvard Medical School, Boston, MA; University of Texas Southwestern, Dallas, TX; University of Texas Southwestern/Parkland Hospital, Dallas, Texas; University of Southern California, Los Angeles, CA; UW Medicine Regional Burn Center, Harborview Medical Center, Seattle, WA; Spaulding Rehabilitation Hospital, Boston, Massachusetts; Massachusetts General Hospital/Shriners Children's, Boston, MA; Spaulding Rehabilitation Hospital, Charlestown, MA; University of Washington, Seattle, WA; Spaulding Rehabilitation Hospital, Harvard Medical School, Boston, MA; University of Texas Southwestern, Dallas, TX; University of Texas Southwestern/Parkland Hospital, Dallas, Texas; University of Southern California, Los Angeles, CA; UW Medicine Regional Burn Center, Harborview Medical Center, Seattle, WA; Spaulding Rehabilitation Hospital, Boston, Massachusetts; Massachusetts General Hospital/Shriners Children's, Boston, MA; Spaulding Rehabilitation Hospital, Charlestown, MA; University of Washington, Seattle, WA; Spaulding Rehabilitation Hospital, Harvard Medical School, Boston, MA; University of Texas Southwestern, Dallas, TX; University of Texas Southwestern/Parkland Hospital, Dallas, Texas; University of Southern California, Los Angeles, CA; UW Medicine Regional Burn Center, Harborview Medical Center, Seattle, WA; Spaulding Rehabilitation Hospital, Boston, Massachusetts; Massachusetts General Hospital/Shriners Children's, Boston, MA

## Abstract

**Introduction:**

Voice plays a prominent role in verbal communication and social interactions. Acute burn care often includes intubation, mechanical ventilation and tracheostomy, which may impact voice quality. However, the issue of long-term dysphonia remains underexplored. Thus, this study aims to examine the frequency of and associated factors with voice changes long term in the burn population.

**Methods:**

Adult data from a multicenter longitudinal database from 2015-2023 were analyzed. The frequency of self-reported vocal changes (yes/no) was examined at discharge and 6, 12, 24, and 60 months after injury. Demographic and clinical data were compared between those with and without vocal changes at 12 months after injury. An omnibus chi-square test examined the incidence of voice changes among burn survivors based on ventilator days. Additionally, the percentage of tracheostomies by ventilator days was observed and compared amongst those with and without a voice change. Logistic regression analysis examined the associations between demographic and clinical factors and self-reported voice change at 12 months.

**Results:**

A total of 582 individuals with burn injuries were included (65 with and 517 without voice change at 12 months). Voice changes were most commonly reported at discharge (16.4%) and persisted over the 60-month follow up period (11.6-12.7%). Those with voice changes were more likely to have had a flame burn, inhalation injury, tracheostomy, outpatient speech-language pathology, head/neck burn, larger burn size, mechanical ventilation, more ventilator days, and multiple trips to the operating room than those who reported no voice changes (p < 0.001). Increasing frequency of voice changes is associated with greater ventilator days (p < 0.001). For those on a ventilator more than 21 days, 48.7% experience voice changes at 12 months and 83.3% had received a tracheostomy. The regression analysis demonstrates that individuals that were placed on a ventilator and received a tracheostomy were more likely to report a voice change at 12 months (284%, p=0.027 and 431%, p< 0.001, respectively).

**Conclusions:**

Voice changes were frequently observed in people living with a burn injury, showing that symptoms can persist for up to five years after injury. Mechanical ventilation and tracheostomy were the primary factors associated with persistent voice changes after injury. Future studies could explore the impact of speech-language pathology and other interventions on individuals experiencing long-term dysphonia following burns.

**Applicability of Research to Practice:**

This study emphasizes the need to understand the long-term voice effects of intubation and tracheostomy in burn care.

